# Editorial: COVID-19, Aging, and Public Health

**DOI:** 10.3389/fpubh.2022.924591

**Published:** 2022-06-13

**Authors:** Emily J. Nicklett, Marcia G. Ory, Kimson E. Johnson, Tzvi Dwolatzky

**Affiliations:** ^1^Department of Social Work, College for Health, Community and Policy, University of Texas at San Antonio, San Antonio, TX, United States; ^2^Department of Public Health, College for Health, Community and Policy, University of Texas at San Antonio, San Antonio, TX, United States; ^3^Center for Population Health and Aging, School of Public Health, Texas A&M University, College Station, TX, United States; ^4^Department of Health Management and Policy, School of Public Health, University of Michigan, Ann Arbor, MI, United States; ^5^Department of Sociology, University of Michigan, Ann Arbor, MI, United States; ^6^Ruth and Bruce Rappaport Faculty of Medicine, Technion - Israel Institute of Technology, Haifa, Israel

**Keywords:** public health, aging, gerontology, geriatrics, COVID-19, ageism, social determinants of health, risk perceptions and coping

## Introduction

COVID-19 has had especially detrimental effects on older adults, who have disproportionately experienced severe complications, hospitalizations, and mortality as a result. The public health response has noted the vulnerability of older adults in these ways, but less is known about how older adults perceive their risks, follow recommended guidelines, interact with family and friends, negotiate health care and social services, and navigate their home and community environments. Further, there is limited information about differences in these experiences within and between populations or the successes and challenges of public health professionals and systems to address these concerns, especially from an international perspective.

The “*COVID-19, Aging, and Public Health*” Research Topic addresses this knowledge gap by including contributions on public health and ageism, health care and social service responses to COVID-19, health equity/social determinants of health, social isolation and social support, risk perceptions and coping, and active aging and health-related behaviors during the COVID-19 pandemic. The Research Topic incorporates a range of article types to inform health and aging research, practice, and policy strategies, including brief research reports, original research articles, systematic reviews, general commentaries, opinion and perspective pieces, and policy briefs. The Research Topic also underscores the broad geographic scope of aging and public health research, with contributions from Asia (China, Hong Kong, Vietnam, Thailand), Europe (Italy, France, Luxembourg, Portugal), the Americas (Brazil, Ecuador, Canada, United States), and the Middle East (Israel and Saudi Arabia).

Highlighting six salient themes around major COVID-19, aging, and public health issues, this Research Topic draws insights from the 40 articles in this collection into current public health impacts and responses.

## Research Topic Contributions

### Theme 1. Public Health and Ageism

The Research Topic begins with identifying ways in which the spread of—and protections against—COVID-19 exacerbated problems of ageism within and across societies. In their longitudinal study, Kornadt et al. examine multidimensional perceptions on aging and perceived ageism among community-dwelling residents in Luxembourg during the COVID-19 pandemic. The authors discovered that targeted experiences of ageism during the crisis negatively impacted older adults' self-perceptions of aging and may have long-term consequences for older adults' development. Beyond the COVID-19 pandemic, raising knowledge of the nature and consequences of ageism may help develop measures to counteract negative consequences for older adults. A related article by Lagacé et al. examines the extent to which older adults' voices and perspectives were included in public discourse among Canadian Francophone adults using content analysis of two French Canadian media op-eds and comment pieces published during the first wave of the pandemic. The authors find that older people were relatively absent from this discourse, which framed older adults passively as people who need to be ‘fought for'—rather than to ‘fight along with'. Indeed, the study by Barth et al. was among the first to assess the role of ageism in the COVID-19 pandemic and protocols by interviewing older adults themselves in an urban area of France. The authors reported that experiences of age-based discrimination and ageist attitudes in public narratives and within family networks were prevalent—and might have increased—during the COVID-19 pandemic. Social isolation is highlighted as a particular consequence of concern.

The pandemic shed light on the lack of governmental support and inadequate funding for public health systems, which were ill-prepared to address major public health crises such as COVID-19. In an opinion piece, Fried reimagines a modernized integrated public health approach that aligns public health systems with community needs, especially for the most vulnerable community members. Although Fried references the US context, her message regarding the urgent need for collective public health action to prevent the spread of infection while mitigating the untoward effect of unintended social isolation especially damaging to older adults has relevance globally. Similarly, Morrissey and Rivera-Agosto underscore the critical role public health can play to protect vulnerable older adults during public health emergencies. Recommending closer dialogue across aging, public health, and legal sectors, two public health lawyers share their first-hand perspectives on the role of civil society in influencing policy decision making, advocating for legal and ethical reforms, and social change by working collaboratively with the New York State Bar Association. Resultant recommendations will help create a robust public health response emphasizing values of equality, equity, adequacy, and justice for all persons impacted by the pandemic.

Several manuscripts highlighted specific country challenges, experiences, perspectives, and best practices in addressing the COVID-19 pandemic. Effective public communication about the facts and myths associated with COVID-19 is critical for engaging the public in appropriate preventive measures to stop the disease spread. Alanezi et al. examine public knowledge in Saudi Arabia about COVID-19 symptoms, treatment, transmission, information types and sources, and promotional channels in Saudi Arabia. While most respondents had a good basic understanding of the risks of and treatments for COVID-19, a significant minority were ill-informed or did not follow stay-at-home recommendations. These findings guided the authors to develop a framework for public awareness during the COVID-19 outbreak to inform government-sponsored public health campaigns.

Combining public health and geriatric medicine approaches is seen as a viable solution to protecting older adults during the COVID-19 pandemic. Clarfield and Dwolatzky provide an overview of Israel's “first mitigate, then eradicate” strategies that provide important public health lessons for other counties. They comment on the successes and challenges of initiating a national program for nursing home residents as well as the rollout of a population-based vaccine program. Acknowledging that the pandemic is evolving both geographically and temporally, the authors call for attention to ethical and socioeconomic considerations in the treatment of COVID-19 world-wide that protect the most vulnerable community members without perpetuating ageist attitudes. Reflecting on a previously published *Frontiers* article calling for a new model of care to address the fragility of public health services in Europe (1), Kuo and Trejo call for greater attention to system-level changes such as investing in workforce development to recruit and retain an adequate aging services workforce. Echoing other authors in this Research Topic, they also recommend a new model of care based on multi-sector collaboration that cuts across traditional care boundaries. They cite the emergent successes of the age-friendly cities and community movements to meet the health and social needs of older adults in Los Angeles, California, and pose as a model for care in the U.S. and other countries as well.

### Theme 2. Health Care and Social Service Responses

COVID-19 has involved new constraints for providing health care services to older adult populations, but also new opportunities. For geriatricians, the first wave of COVID-19 was associated with great uncertainty. Clear health policy directions had not yet been determined and the older population has been particularly vulnerable to poor outcomes and to a stigmatic ageistic approach. Recognizing the urgent need for leadership, Dwolatzky challenges geriatricians to step forward and take the lead in developing policy to protect older people from exposure to the virus and to ensure the provision of humane medical care and support. As newer variants of the virus continue to create additional waves of COVID-19, the challenge posed by this manuscript remains timely and relevant.

Frailty is now recognized as a key geriatric syndrome, indicating vulnerability of the older person to disease and poor outcomes. In an interesting association between frailty of the individual and frailty of the healthcare system, Crosignani et al. describe how the stress of the COVID-19 pandemic revealed severe inherent weaknesses in the structure, priorities, and organization of the Italian healthcare system. They emphasize the need to move away from a hospital-centered model that failed to provide care for the older frail population to a community-based multidisciplinary person-centered service.

Comprehensive geriatric assessment has been clearly shown to improve the medical care and outcomes of older patients. Recognizing that many older patients were being admitted to non-geriatric wards as the COVID-19 pandemic struck Belgium, Angioni et al. decided to provide these wards with the support of a multidisciplinary mobile geriatric team (MGT). Relating to Intrinsic Capacity (IC) based on cognition, mobility, vitality, mood and sensory domains, the authors believe that MGT was able to emphasize a more holistic approach to the older patient, promote better outcomes, and encourage decision-making based on comprehensive assessment rather than merely relating to chronological age. For geriatric oncologists, the COVID-19 pandemic has highlighted the concept of governance, both on a national as well as an international level. Fonseca et al. discuss the multi-level structure of governance as it relates to the pandemic in Portugal. While the classic “top-down” structure of input from the World Health Organization, European authorities, and national government is usual, the authors raise the importance of also involving medical personnel and professional societies in determining policy to provide adequate care for older cancer patients during the pandemic.

The COVID-19 pandemic has also introduced constraints on the health care workforce for the provision of care, diagnostic processes, and assessment strategies for older adults. Looking at the use of emergency medical services (EMS) among Latinos aged 50 years and older in California counties, Melgoza et al. find that while respiratory distress related EMS calls among Latinos were lower prior to the pandemic, this increased during the first wave of COVID-19 compared to non-Hispanic Whites. The authors discuss the racial and ethnic differences observed in their findings and raise important issues relating to EMS health disparities. There is also a clear need to strengthen healthcare services provided to older residents of nursing homes and skilled nursing facilities (SNF), which have experienced high rates of COVID-19 morbidity and mortality. Levy-Storms et al. present recommendations for Certified Nurse Aid (CNA) training based on data reported by CNAs as well as U.S. government data. They emphasize the importance of providing CNAs training to reduce health risks from infectious diseases and to improve how they relate to SNF residents during care. Such training can help prepare the front-line workforce for future public health emergencies.

Focusing on diagnostic processes and assessment, the vulnerability of the older population to COVID-19 infection and its sequelae emphasizes the importance of early detection and screening. Van Son et al. correctly point out that measuring temperature has limited value in older adults and that the atypical presentation of COVID-19 may delay diagnosis and therapeutic intervention. An important practical observation is that “silent hypoxemia” should be sought and documented, and that this measurement should be available for those older people at home or in senior-living facilities. Acute kidney injury (AKI) is also an important condition in older people, especially for those who are frail, and it is associated with poor outcomes. Chuang et al. used the DEMATEL approach to identify the following risk factors for AKI, namely comorbidity, malignancy, diabetes, creatinine, estimated glomerular filtration rate, and nutritional assessment. Based on these factors, the authors encourage the development of a structured index for predicting AKI especially faced with high COVID-19 related morbidity.

COVID-19 has also imposed unique challenges and opportunities for practitioners and agencies providing social services for older adult populations. Elder abuse and end-of-life palliative care illustrate this point. Adult abuse at times of crisis is often more prevalent, and this is the case with the COVID-19 pandemic and associated lockdown, with the closure of senior centers and more limited home care services. Liu and Delagrammatikas describe the impact of the pandemic on Adult Protective Services (APS) in the United States and describe the difficulties encountered in serving older and dependent abuse clients. They also report the welcome decision to provide federal funding to support APS programs. Quality end-of-life care requires clear direction regarding practitioner decision-making promoting autonomy and personal preferences. Nguyen et al. assessed the degree of provision of advance care planning (ACP) directives, the determination of a healthcare proxy (HCP), and attitudes toward ACP among adults older than 50 years living with HIV during the COVID-19 pandemic. While the majority of respondents reported having an ACP or HCP, most believed an ACP to be more important now at the time of the pandemic.

### Theme 3. Health Equity and Social Determinants of Health

The impacts of the COVID-19 pandemic are not equally spread in society and older adults who are racial and ethnic minorities and socioeconomically disadvantaged are particularly hard-hit. Guerrero and Wallace employ the World Health Organization's Health Inequity Causal Model to examine how numerous social determinants of health put U.S. older adults of color at greater risk of poor COVID-19 outcomes. The authors strongly encourage future equity-focused solutions to the epidemic focus on the most vulnerable populations who are at greater risk for differential exposure, vulnerability, and inequitable consequences. They emphasize, however, that a commitment to long-term health equality work is necessary to promote equity in areas of multiple social determinants of health (i.e., housing, education, labor force safeguards, and income) to support future health equity for all. Regarding socioeconomic disparities, Bergeron et al. review the disproportionate physical and mental health consequences faced by lower-income older adults. They then suggest practical strategies for governments, communities, and organizations to provide opportunities for low-income older adults to engage in health-promoting behavior.

These racial/ethnic and socioeconomic disparities are consequences of deeply embedded structural inequities at societal levels. In a perspective piece, Lee highlights the interplay between six COVID-19 amplifiers, health inequity triggers, and existing social inequity among the U.S. older adult population. Emerging vulnerabilities shed light on the ramifications of recent ageist policy responses to COVID-19 and the necessity to find cost-effective policies that work for older adults within present budget restrictions. Exploring different anticipated pathways that account for differing mechanisms of social determinants on health inequality can aid in developing interventions that account for complex, linked, and cascading factors on health inequity among older adults.

In the context of frontline essential workers with high COVID-19 exposure, Ma et al. draw attention to the persistent structural inequities in social determinants of health and the historically racialized immigration system, which contribute to COVID-19 mortality and barriers to care among older Asian Americans in the U.S. Asian immigrants have been prevented from qualifying for public aid, such as COVID-19 testing and immunization programs, due to the fear of a “public charge” regulation. To promote the health and wellbeing of older Asian Americans, the authors advocate for racial/ethnic data disaggregation and meaningful engagement of older Asian Americans in research and policy with a commitment and investment in multi-sectoral collaborations.

COVID-19 infections in nursing homes brought widespread media and policy attention, but the disproportionate impact of COVID-19 deaths in high-minority nursing homes warrants specific examination. Weech-Maldonado et al. examine whether the racial/ethnic composition of residents in nursing homes is associated with the level of COVID-19 mortality among residents. As the pandemic progresses, nursing homes that serve primarily minority populations have revealed the devastating consequences of existing racial/ethnic imbalances in minority communities during the COVID-19 epidemic. Policy interventions should focus on the lack of resources for nursing homes that serve predominantly Black and Hispanic nursing home residents and address the systemic inequities, existing healthcare disparities, and social inequalities inside nursing home communities.

### Theme 4. Social Isolation and Social Support

A discussion of the toll of COVID-19 on older adults would not be complete without addressing the high social costs of isolation. In a research article, Adepoju et al. investigates numerous indicators of social isolation among community-dwelling older individuals in the U.S. and variations between two overlooked groups: African American and Hispanic older adults. The unintended health-related consequences associated with social isolation during the post-pandemic period underscore the importance of identifying ways to minimize the potential long-term impacts of COVID-19 on physical and mental health. The authors find that social isolation affects older persons in various ways and that culturally informed activities are needed to address the potential effects of social isolation among racially minoritized populations.

In an opinion article, Heymann characterizes how older adults are, in some circumstances, “doubly punished” by the COVID-19 pandemic: first, in terms of experiencing the highest rates of COVID-related mortality, and second, in terms of lockdown conditions. Focusing on older adults in nursing homes, the author emphasizes the dire need to consider and address the personal and social costs of social isolation in care homes. Drawing on their Gero-COVID initiative, Coin et al. examines how quarantine affected the psychological wellbeing of older adults in Italy with cognitive impairment. Those with more severe cognitive impairment had worse outcomes in terms depression and anxiety, attributed to poorer coping skills. In addition to concerns about physical health symptoms, health care professionals need to be aware of psychological distress experienced both by persons with cognitive impairments as well as their caregivers during periods of social distancing.

Two original research articles examine the impact of COVID-19 on life-space, daily life, and social interactions of community-dwelling older adult populations. Focusing on Brazilian older adults, Perracini et al. investigates the immediate impact of social restrictions due to the COVID-19 pandemic on life-space mobility and corresponding health behaviors and outcomes. Their findings underscore the importance of developing comprehensive strategies to limit pandemic repercussions and utilizing innovative digital technology to deliver physical activity and rehabilitation programs to older persons. The authors call for further action to address the decline in life-space mobility among the most vulnerable older adults. The novel examination of methodologies for tracking patterns of time use and social contacts can shed further light on populations at risk. Focusing on older adults in the U.S., Chen used hurdle regressions of pre-pandemic time use data and find that older age was associated with less time spent in public places, less time spent with family, but more time spent with non-family members. This study can help identify risk factors related to social isolation and potential exposure to COVID-19.

In addition to examining the impact of COVID-19 on social isolation of older adults, two articles examine potential strategies to promote social interaction and social support during the pandemic: Memory Cafés and companion animals. Memory Cafés, according to Masoud et al., are effective facilitators of social connectedness for people living with dementia and their family care partners in Texas, providing opportunities to socialize in a supportive environment. When in-person gatherings were restricted in COVID-19, virtual Memory Cafés provided regular online social engagement opportunities, including in geographically marginalized and underserved areas. The authors' findings suggest that virtual Memory Cafés provide opportunities to participate in cognitively challenging activities and connect to community resources. Companion animals can also be a source of social support but might also bring unique challenges during the COVID-19 pandemic. Applebaum et al. examine data from a large survey of U.S. pet owners to determine the impact of pet ownership on the health and well-being of older adults. They find that older adults were generally less lonely—despite reporting lower levels of support—than younger groups. The authors identified pros (e.g., company, support, stress relief, exercise) as well as cons (e.g., veterinary care access, obtaining supplies, financial concerns) of pet ownership as it relates to the COVID-19 pandemic.

### Theme 5. Risk Perceptions and Coping

Six articles in the Research Topic contribute to the understanding of risk perceptions and coping during the COVID-19 pandemic. With specific regard to risk perceptions, protection measures such as social distancing and mask-wearing can help prevent the spread of COVID-19. Stay-at-home mandates were a major public health recommendation to help protect older adults early in the pandemic before the widespread availability of vaccines. Macy et al. analyzed data from a nationally representative US survey to examine older adults' beliefs underlying their decisions to stay home as recommended by governmental executive orders. This study revealed several interpersonal, mental health, and leisure/recreational facilitators for older adults' intentions to stay home. The authors also identified concrete intervention strategies to help older adults engage in recommended public health actions including self-efficacy building interventions and appropriately tailored health communication messages.

In addition to social distancing, face mask-wearing is a major precautionary measure to stop the spread of SARS-CoV-2. However, little is known about the psychological correlates and consequences of mask-wearing. Kwan et al. examine the relationships between face-wearing behaviors, health beliefs, and depressive symptoms among older people in Hong Kong, a country with a pre-COVID 19 tradition of mask-wearing. Health beliefs about disease severity and efficacy of preventive measures were associated with face mask use. However, face mask reuse was associated with greater depressive symptoms among those with greater perceived severity and inadequate cues to preventive measures. This study points to the complexities involved in understanding the full context of specific recommended preventive health measures, and the importance of co-occurring mental health supports. The extant literature has identified populations at higher risk of more severe clinical symptomology associated with SARS-CoV-2, but the public may perceive risks differently.

Older adults with chronic health conditions experience heightened risks of mortality and adverse COVID-19 outcomes. Aumala et al. examine the perceived risk of infection and complications in people with hypertension living in Ecuador. While adults with hypertension in outpatient settings may be aware of risks, there is a need for health systems to educate their patients about the appropriate use of protective measures to mitigate personal risks and disease spread to others. While necessary to prevent the spread of COVID-19, protection measures such as social distancing can also entail a high social cost. Persons living with dementia are traditionally viewed as a particularly vulnerable group, and the COVID-19 pandemic may amplify vulnerabilities.

Most of the studies in this Research Topic assessing the impacts of the COVID-19 pandemic are quantitative in nature. Qualitative studies are valuable for providing insights into perceptions of COVID-19, available resources, coping styles, and predictors of overall emotional and physical health. This collection includes three qualitative studies adding depth to quantitative research studies.

In the first qualitative study, Goins et al. employ qualitative methods to provide an in-depth view of how older adults in the United States are responding to COVID-19 in the early stages of the pandemic. Topics of importance to participants reflect four main themes: (1) risk perceptions; (2) financial impact, (3) coping and (4) emotions, which resonate with other quantitative research endeavors. While many older adults showed resourcefulness in coping using both problem-focused and emotion-focused strategies, having low-to-no cost existing resources to bolster mental health during social isolation is highly recommended. Older adults face many challenges and stressors related to the COVID-19 pandemic, but they also could draw upon behavioral and emotional coping strategies to address these challenges.

In the second qualitative study, Finlay et al. examine qualitative data from an online multi-frame study of older-aged 55 and older in the United States. Through qualitative content analysis, the authors find that frequently reported strategies included health-limiting approaches (e.g., over-eating), but that most participants reported health-promoting (e.g., exercising and going outdoors, following public health guidelines, modifying routines, adjusting attitudes, and staying socially connected) strategies.

In the final qualitative study of this theme, Greenwood-Hickman et al. examine a Zoom-based intervention to target sedentary behaviors among older adults during the COVID-19 pandemic. While most participants reported increases in sedentary behavior during the pandemic, many also reported higher levels of outdoor or online physical activity. Participants also characterized virtual connection *via* phone and video to help with social connection, engagement, and cope with stressful pandemic circumstances.

### Theme 6. Active Aging and Health-Related Behaviors During the Pandemic

The COVID-19 pandemic has disproportionately affected older adult populations in terms of severe morbidity and mortality, but also affects opportunities for older adults to engage in health-promoting behavior, such as engaging in physical activity and maintaining a healthy diet. The sixth theme includes four articles that examine health-promoting behavior and active aging in the context of the COVID-19 pandemic. As part of a longitudinal web-based survey, Joseph et al. examine physical activity data before and during the COVID-19 pandemic among adults aged 50 and older. The authors find that physical activity levels declined and remained below pre-pandemic levels among participants. They recommend strategies to promote safe opportunities for middle-aged and older adults to engage in physical activity when social distancing is needed. There has also been limited understanding of the impacts of COVID-19 on the ability of older adults to access food and maintain healthy diets. Nicklett et al. conducted a scoping review of the literature to characterize changes in food access, diet quality, and nutritional status among middle-aged and older adults during the COVID-19 pandemic. Using a socioecological model approach, they identified singular (e.g., intrapersonal and environmental) and hybrid spheres of influence (e.g., intrapersonal/environmental) on the food environment. While most studies reported challenges to food access and/or poorer diet quality, especially among the most vulnerable populations, the authors concluded that more research is needed that examines the impact of the pandemic on food access and security and how these barriers differ among older adult populations.

There is a need to look downstream as well as upstream for strategies to promote health behavior and active aging in the time of COVID. When looking at health promotion strategies, health literacy could play an important role in health behavior and health outcomes of older adults during the COVID-19 pandemic. In a cross-sectional study conducted at outpatient departments in hospitals and health centers in Thailand and Vietnam, Do et al. examine differences in health literacy, depressive symptoms, dietary behavior, and physical activity between adults with and without suspected COVID-19 symptoms. They find that in older adults with COVID-19 symptoms, those with higher health literacy were more likely to engage in physical activity, eat healthier diets, and were more likely to experience depressive symptoms.

On a broader level, health-promoting behavior and active aging should be part of national and international strategies, complete with metrics, goals, and priorities. In their policy brief, Costa et al. argue that The Decade of Healthy Aging 2021-2030 and its baseline report, the 2018 Active Aging Index Analytical Report provide a model to discuss goals and priorities around healthy aging in Portugal and more broadly in other European counties. The authors emphasize the importance of aligning national approaches (Portugal's National Strategy for Active and Healthy Aging) with European Commission and international (World Health Organization) approaches for the collection and analysis of comparable data nationally and internationally. These recommendations are relevant for mobilizing a worldwide effort to promote global healthy aging.

## Conclusion

This collection of 40 articles broadly examines the impacts of COVID-19 on older adult populations, as well as future directions in research, policy, and practice. As we are embarking on the third year of the COVID-19 pandemic, it is important to understand the evolution of the disease, as well as changing public health responses. Our hope is that lessons learned in the first 2 years from various geographic regions and populations can help mitigate the worldwide effects on older adults, their families, and communities.

## Author Contributions

EN, MO, KJ, and TD contributed to the design, organization, and writing of the manuscript. All authors contributed to the manuscript revision, read, and approved the submitted version.

## Funding

Research reported in this publication was supported by the National Institute on Aging of the National Institutes of Health under Award Number T32AG000221.

## Author Disclaimer

The content is solely the responsibility of the authors and does not necessarily represent the official views of the National Institutes of Health.

## Conflict of Interest

The authors declare that the research was conducted in the absence of any commercial or financial relationships that could be construed as a potential conflict of interest.

## Publisher's Note

All claims expressed in this article are solely those of the authors and do not necessarily represent those of their affiliated organizations, or those of the publisher, the editors and the reviewers. Any product that may be evaluated in this article, or claim that may be made by its manufacturer, is not guaranteed or endorsed by the publisher.
